# Foot biomechanics- emerging paradigms

**DOI:** 10.1186/1757-1146-7-S1-A1

**Published:** 2014-04-08

**Authors:** Stephen F Albert

**Affiliations:** 1 Chief Podiatric Section, Surgical Service. Dept. of Veterans Affairs Medical Center, Denver, Colorado 80220, USA

## 

Too many times theories of how the human foot functions and therefore how mechanically inducted foot problems are treated have been presented as if they were facts. The dogmatic adherence that sometimes ensues from such an approach has frequently stifled the evolution of foot mechanics. This has been particularly apparent in the field of podiatry which has been dominated by the Root paradigm. Briefly, the Root paradigm proposes that the human foot functions ideally around the subtalar joint’s neutral position. Additionally, the forefoot to rearfoot frontal plane relationship ideally should be perpendicular. Furthermore, deviations from those ideal positions are termed deformities. [1,2]

Biomechanical treatments according to Root are intended to re-align the foot so as to function around the neutral subtalar position and/or prevent frontal plane compensations from a deformed forefoot. In essence this paradigm is based on foot morphology.

Several studies have raised doubts as to the validity of the Root paradigm. [3-8] This abstract is a review of several papers which raise those doubts and explores emerging paradigms of human foot function which align more with current research findings on foot function.[9-11]
